# Supplementary material for: Are carbon emissions trading and green financial instruments synergistic? -Comprehensive quantitative research based on content analysis
Source: PLoS One. 2024 Mar 7;19(3):e0298601. doi: 10.1371/journal.pone.0298601 (PMC10919601; doi:10.1371/journal.pone.0298601)
Supplement: S1 Data — (ZIP) [file pone.0298601.s001.zip › Data/地级市Z_F工作报告.zip!/║■─╧/│ñ╔│╩╨/└┤╫╘╣1⁄2╓┌║┼í╛┬φ┐╦╩2╛▌═°í┐_2005.docx]

各位代表：

　　现在，我代表市人民政府向大会报告工作，请予审议。并请各位政协委员和其他列席人员提出意见。

一、2004年政府工作回顾

　　过去的一年，在市委的正确领导下，我们紧紧依靠全市人民，坚持以科学发展观为指导，认真落实国家宏观调控政策，更加突出经济增长的质量和效益，更加突出社会事业发展，更加突出提高人民生活水平，奋发图强，锐意进取，圆满地完成了市十二届人民代表大会第二次会议提出的各项目标任务。全年实现地区生产总值1108.07亿元（初步测算，下同），增长14.8%；完成地方财政收入104.36亿元，增长31.6%；完成全社会固定资产投资668.09亿元，增长38.9%；城乡居民人均可支配收入分别达11021元和4154元，增长11%和13.9%；城市综合实力跻身全国百强城市第16位。长沙开始步入了经济社会与生态环境相协调，综合实力明显增强的良性发展轨道。

　　（一）产业结构调整成效显著，工业增长实现提速增效。工业主导地位不断加强，全年实现工业总产值1006.06亿元，增长25.2%；实现工业增加值329.53亿元，增长19.9%，工业对经济增长的贡献率达41%。产业集群度提高，两区六园实现工业总产值334.95亿元。支柱产业和骨干企业规模不断扩大，实现规模以上工业总产值706.06亿元，增长28.3%。十大标志性工程完成工业增加值155.08亿元，占规模工业的62%。产值过亿元企业达97家，长沙烟厂实现销售收入过100亿元。实现高新技术产业总产值500亿元，增长28%。市属国有工业企业改革任务基本完成。四县(市)完成规模以上工业总产值343.99亿元，增长29.8%。全市规模以上工业实现利润50亿元，利税率居全国44个中心城市第一位。

　　（二）农业政策效应明显，农村发展取得历史性突破。认真贯彻中央一号文件，落实各项农业政策直补资金7848.85万元，降低农业税税率3.97个百分点，减少农业税和农业特产税1.3亿元。农民种粮积极性空前高涨，粮食生产实现了面积、单产、总产、效益大幅度提高，实现农业总产值172.07亿元，增长7.3%。农业产业带建设初具规模，长沙跻身全国“十大苗木之乡”行列。健全了动物防疫体系，启动了农业标准化生产和无公害农产品基地建设。新建农民工廉租房5万平方米。全年完成农村劳动力培训6.08万人，外出务工人员达120.25万人，实现劳务总收入53.16亿元。23个农村小康示范村建设开局良好。农村各项改革取得了显著成效，全市农村呈现出蓬勃发展的喜人景象！

　　（三）统筹城乡规划建设，城乡环境明显改善。城市各类详规和专业规划编制工作力度加大，县域城镇体系规划和乡镇总体规划基本完成。城市建设继续推进，重点工程完成投资90亿元，新增城市道路72公里。小城镇建设步伐加快，投入资金45.3亿元，完成建设项目500个。新建和改造县乡公路463.5公里。城管体制进一步理顺，管理水平不断提高。第一期背街小巷改造提质和站厕建设顺利完成。新建城市林业生态圈25.9公里，城区新增绿地195.8公顷。岳麓区、长沙县、望城县通过国家级生态示范区验收。明阳山殡仪馆建成并投入使用。公用事业和市直管公房市场化改革取得实质进展。城市勘测和国土资源管理工作均获全国先进单位，水利建设再夺全省“芙蓉杯”。长沙县获中国人居环境范例奖。

　　（四）商贸流通繁荣兴旺，对外开放全方位互动发展。全年实现社会消费品零售总额525.13亿元，增长16.2%。物流配送、金融保险等新兴服务业和住房、汽车等消费大幅增长，商品房销售面积达519.95万平方米，增长59.6%。完成旅游收入138亿元，增长17.6%。积极参与“湘洽会”，成功举办“港洽会”，全年举办各类节会150余个。引进外来资金347.46亿元，其中实际利用外资5.01亿美元。实现进出口总额24.54亿美元，增长20.1%。区域合作开创了新局面，长株潭经济一体化进入项目实施新阶段，泛珠三角合作有了新的开端。成功举办了“国际友好经贸合作洽谈会”、“世界旅游小姐年度皇后大赛”等具有国际影响的大型活动。长沙的知名度、美誉度和对外影响进一步扩大。

　　（五）民主政治建设稳步推进，社会大局和谐稳定。充分尊重人民群众的政治权利，自觉接受人民代表大会及其常委会的监督，积极支持人民政协发挥民主协商、参政议政作用。全年办理人大议案和人大代表建议批评意见215件，办理政协委员提案324件。基层民主政治建设迈出新步伐，村支两委干部交叉任职和直选试点工作取得成功，村级区划调整基本完成。建设宣传文化法制中心(站)153个。认真贯彻《行政许可法》，加强了行政执法和政务公开工作。制定和废止政府规章9件，提请市人大常委会制定、修改和废止地方性法规11件。进一步改革和完善了市政府工作机构。政府规划能力不断加强，位列全国大中城市第7位。建立了集中处理信访突出问题及群体性事件联席会议制度，解决了一批群众反映突出的问题。强化社会治安综合治理基层基础工作，不断完善“严打”整治斗争长效机制，创建“平安长沙”初见成效。

　　（六）社会各项事业全面进步，人民生活水平有所提高。科技产业基地和科技推广普及工作取得新的成绩，加入了世界科技城市联盟。全市申请专利3662件，形成了一批核心技术和自主知识产权成果。各类教育协调发展，学校布局调整和薄弱学校改造成效明显，素质教育和农村教育得到加强，长沙大学升格为本科院校。率先在中西部地区实行人才居住证制度。文化体育和广播电视事业稳步推进，成功举办了中韩国际足球赛和第四届全国小品大赛。群众文化与全民健身运动蓬勃开展。初步建立了公共卫生突发事件的应急机制。农村中心卫生院建设取得初步成效。天心区率先通过全国社区卫生服务示范区验收。全市新增城镇就业人员8.62万人，实现再就业4.99万人，我市被国务院授予“全国再就业先进集体”。全年共投入社会保障补助、社会福利救济和抚恤资金8.93亿元。出台并实施了工伤、生育保险等办法。人口和计划生育工作进一步加强，再获全省计生模范市称号。投入38.9亿元全面完成了省、市八件实事，切实履行了对人民群众的庄严承诺。统计、审计、物价、工商、税务、质量监督、药品监督、海关、人防、民族宗教、台办、新闻出版、档案、市志、消防、电力、气象、地震、水文、工会、共青团、妇联、残联、关协、侨联、老龄委等工作都取得了新成绩。

　　各位代表，2004年是长沙经济和社会发展实现历史性跨越的一年；是“三个文明”协调发展，人民生活水平明显提高的一年；是加强区域合作与交流，对外影响更加扩大的一年。这些成绩的取得，是市委正确领导的结果，是市人大、市政协有力支持和监督的结果，凝聚了全市人民的辛勤劳动和聪明才智。在此，我代表市人民政府向全市人民、驻长解放军、武警官兵和政法干警，向各民主党派、工商联、无党派人士和各人民团体，向所有关心和支持长沙建设与发展的各界朋友表示衷心的感谢，并致以崇高的敬意！

　　历史的经验值得重视。过去一年的工作实践，我们认为有以下几点值得思考和深化：一是注重调整经济结构，提高国民经济的自主增长能力。在宏观经济调控的大背景下，我们突出工业化带动城市化和农业产业化，着力推进投资体制改革，进一步支持加快发展非公有制经济，扩大非政府资本特别是企业自主投资比重；加强政策扶持，扩大产业投资特别是工业投资的比重，为经济平稳快速发展增强了后劲。二是注重统筹城乡发展，不断增强农村发展活力。在落实中央一号文件中，我们进一步提高了粮食综合生产能力和农民增收水平，促进了农业产业化进程；加大了财政转移支付力度，增强了扶贫帮困功能，加强了农村基础设施建设；始终坚持把农业、农村、农民工作作为重中之重，为建设全面小康社会夯实了基础。三是注重严格保护土地资源，切实加强环境保护。在落实国家“实行最严格的土地政策”中，我们进一步加大了盘活存量土地的工作力度，对基本农田实行最严格的保护，确保重大经济和社会发展项目用地，着力提高土地投资密度和产出效益。加大了生态建设和环境污染治理力度，有效地提高了我市环境质量和可持续发展能力。四是注重促进经济和社会协调发展，切实关注民情民生民利。我们在加快经济发展的同时，更加注重促进社会事业发展。加大了财政对社会保障、科技、教育、卫生、文化、公共安全等社会事业的投入，加强了对困难群体的救助力度，把维护、实现、发展最广大人民的根本利益作为制定政策、开展工作的出发点和落脚点，让改革和发展成果惠及全市人民！

　　各位代表，在总结成绩的同时，我们要充分看到面临的困难和存在的问题：工业支柱产业核心竞争力不强，缺乏重大战略投资项目的支撑，发展后劲不足；电力、资金、能源紧张和原材料、燃料价格上涨，增加了工业经济保持高速高效发展的难度；农村基础设施建设任务繁重，农业产业化程度还不高，农民增收和发展劳务经济有待推出新的举措；城市规划建设，历史文化区街保护，城市管理、污染治理以及生态环境改善，需要做更扎实的工作；政府机关作风、制度建设、行政管理、服务水平有待进一步提高和加强。

二、2005年政府工作的总体目标和主要措施

　　2005年是深入落实科学发展观、全面实现“十五”计划目标、为新一轮发展奠定坚实基础的关键一年。综观当前形势，我们正面临重要战略机遇期。中央实施稳健的财政和货币政策，继续加强和改善宏观调控，国内经济将平稳较快增长，为我市经济的高速高效发展提供了有利的宏观环境；中部崛起战略的实施、泛珠三角区域合作的拓展，为我市发挥区位、资源、生态、人才等综合优势，创造了新的发展空间；我市跻身经济总量千亿元城市行列，聚集资源、承接产业转移、辐射拉动发展的能力大为增强。我们要审时度势，以深邃的世界眼光、争先加压的勇气，乘势而上，在新的一年，把经济和社会发展推上新的台阶。

　　今年政府工作的总体要求是，深入贯彻党的十六届三中、四中全会精神，以科学发展观统领经济社会发展全局，不断调整经济结构，转变经济增长方式，深化改革，扩大开放，突出推进新型工业化进程，以工促农、以城带乡，全面完成“十五”计划，实现经济社会全面协调发展，努力构建社会主义和谐社会。主要预期目标是：地区生产总值增长14%；全社会固定资产投资总额增长20%；社会消费品零售总额增长15.5%；地方财政收入增长15%；城市居民和农村居民人均可支配收入分别增长9%和7%；城镇登记失业率控制在4%以内；人口自然增长率控制在5‰以内。为了完成上述各项预期目标，必须重点做好八个方面的工作：

　　（一）壮大工业经济规模，提高工业增长的质量和效益。一是抓园区工业促进产业集群发展。突出两区六园各自的产业特色和功能，把支柱产业做大做强，促进产业集群式发展。大力发展先进机械制造、电子信息、烟草食品三大支柱产业和十大标志性工程企业，引导资源向优势产业和园区集中。在土地供应、贷款贴息、规费减免、招商奖励、优化服务等方面加大支持力度，重点抓好汽车零部件、工程机械、家用电器等产业的配套。加快生物医药和以新能源为动力的装备制造业的发展。二是抓工业项目带动产能集聚扩张。积极引进战略投资者或合作伙伴，发展混合所有制经济，推动企业做大做强。加强与国内外大企业、大公司的战略联盟，重点发展符合环保要求、产业关联度大、市场前景好的大项目，壮大产业规模。着力提高工业企业的自主投资能力和自主创新能力，抓好玻壳二期、博世电器、长沙电厂、关西涂料、晟通铝箔、燕京啤酒二期、青岛啤酒二期等项目建设。三是抓成长型企业的培育促进中小企业发展。高度重视产业发展的前瞻性研究和规划，着力培育后续支柱产业。加大对品牌企业的扶持力度，鼓励企业技术创新、管理创新和二次创业。建立健全中小企业支撑服务体系，大力培养职业企业家和急需的各类专业技术人才。大力发展非公有制经济和就业型、农产品及资源转化型中小企业，重点抓好100家工业企业增产增效。利用高新技术、先进适用技术改造传统产业，逐步构建低能耗、高产出、循环型产业结构。

　　（二）坚持城乡统筹发展，推动农村结构的战略性调整。一是加强农村公共设施建设。科学编制农业发展规划、镇村公共基础设施规划和用地规划，优化城乡空间结构和资源布局。加快城乡公共设施的对接联网，完善市区到县（市）、县（市）到辖区乡镇“一小时交通圈”，改建县乡公路350公里。完成水利建设投资5亿元，重点抓好五合垸、福安垸、大众垸等防洪整治工程，加强农村水利特别是水库、山塘、沟渠等小水利的建设。加快小城镇建设，增强小城镇吸纳各类生产要素和辐射带动农村经济发展的功能。二是增强农业综合生产能力。加强龙头企业的培育和产业基地建设，壮大农产品流通规模，发展订单农业。推进农业标准化和规模生产，加大农业科技投入和综合开发力度，着力培育品牌产业和产品，增加农产品的附加值。完善农产品质量安全检测体系。支持和鼓励民营资本投资农产品加工业，重点培育一批上规模、带动能力强的龙头企业。积极发展农村专业合作经济组织。深化农村信用社体制改革。三是提升农民的发展能力。继续实施农村劳动力转移培训“阳光工程”，增强农民的创业意识和就业技能，打造劳务品牌，加大劳务输出力度，引导农民向城镇流动。规范农村土地承包经营权流转，严格执行土地征用安置补偿政策，保障失地农民的生产生活来源。抓好农村小康示范村建设。创新扶贫方式，加大扶贫力度。加快乡镇机构改革进程，继续抓好乡镇消赤减债工作。全面落实各项农村政策，从今年开始全市免征农业税。各级各部门要继续不遗余力支持农村，脚踏实地发展农业，一如既往帮助农民，构建解决“三农”问题的长效机制，为全面建设小康农村作出不懈地努力！

　　（三）扩大有效需求总量，促进经济高质高效增长。一是改善投资结构，形成新的投资增量。加快建立市场引导投资，企业自主决策，融资方式多样，中介服务规范的新型投资体制。管好政府投资，对非经营性项目逐步实行代建制。激活民间投资，提高企业自主投资的份额和产业投资的比重。进一步做好创建金融安全区工作。加快推进政府各类投资公司产权多元化的股份制改造。深化完善国企改革，全面加强国有资产监管，实现国有资产的保值增值。二是改造提升传统服务业，扩大有效消费需求。逐步提高城乡居民特别是低收入者收入水平，增强城乡居民的消费能力。加强对全市商业网点规划和建设管理，合理布局大型综合超市，培育和提升大型专业市场，精心打造特色街市。推进中央商务区建设，大力发展楼宇经济。加大旅游资源的综合开发，推进旅游市场化改革，完成旅游总收入160亿元。切实抓好餐饮、文化、休闲、健身等产业的提质改造，保持我市商贸消费在全国的领先地位。三是拓展消费领域，促进新型业态的发展。完善消费信贷等有关政策，鼓励居民购买高档电器、汽车等大额商品。进一步发展房地产二级市场，构建多层次的住房供应、消费、流通体系。加快发展现代物流、金融保险、电子商务、中介服务等新兴服务业，构建区域性综合物流中心和枢纽。开拓农村消费市场，加快发展县城、中心集镇和乡村三级流通市场，加快农村超市和连锁便民店的建设。积极举办和参加国际种业博览会、全国优质稻米博览会，推介长沙产品，提升长沙产品的市场份额。

　　（四）加强城市规划建设管理，构建文化生态型山水城市。一是完善城市功能，加强基础设施建设。充分发挥城市规划在城市建设和发展中的导向和调控作用，突出重点功能区的建设，重点抓好天心生态新城、河西科技新城建设。加快湘江长沙综合枢纽、霞凝港二期、长韶公路、南北火车货站搬迁、引水及水质环境工程等重点项目建设。完成二环线拉通、枫林路改扩、金星大道北段等市政道路建设。抓好长沙电厂、武广客运专线长沙段、长潭西线高速、长株高速等项目的开工建设。做好浏永高速公路建设的前期工作。继续推进背街小巷、城市排水和棚户区提质改造工作。二是推进历史文化名城建设，塑造城市形象。始终注重保护历史文物、历史建筑和文化遗产，突出抓好太平街、化龙池等11条历史街巷和23处名宅的维护和修缮。建设一批爱国主义教育基地。坚持提升现代文化品质和延续历史文脉并重，围绕“一江两岸”，橘子洲、新河三角洲核心区，以新的理念，精心规划设计，把滨江图书馆、博物馆、音乐厅建成标志性建筑。全力打造既能充分表达长沙山水灵秀，深厚文化底蕴，又能满足市民向往，“令居之者忘老，寓之者忘归，游之者忘倦”的新的城市形象景观带。三是加强生态环境保护，建设资源节约型城市。以创建国家园林城市、生态市和卫生市为目标，继续完善城市管理体制，加大城乡结合部的整治力度，积极探索城市维护作业的新模式。开展第二阶段大气污染综合治理。抓好长善垸、岳麓区污水处理厂、八水厂二期和天然气入长综合利用工程。扩大城市绿化覆盖率，加快生态动物园、城市林业生态圈和生态苗圃建设步伐。更加严格地保护土地资源，合理和节约使用土地。发展循环经济和生态环保产业，倡导节约资源，引导绿色消费，努力为全市人民创造一个自然生态良好、文化气息浓厚、舒适优雅有序、承载功能很强的人居和创业环境！

　　（五）积极发展外源型经济，努力提高对外开放水平。一是突出产业和项目招商。各县（市）、行业和园区要集中策划、包装、筛选和储备一批产业、物流业重点项目，主动加强与世界500强、跨国大公司、国内外大型金融机构、上市公司和知名企业的联系。采取项目招商、股权投资、经营权转让、企业并购等方式，吸引更多的大企业、大财团和大的战略投资者来长沙投资兴业。二是创新招商引资方式。坚持政府引导，改进、完善和精简政府组织的大型招商活动，积极推进和组织以企业为主体的专项招商活动，营造园区招商的综合竞争优势。稳步推进全球商务代理、中介招商，促进招商引资由一般性招商向产业招商转变。建立重大招商项目跟进责任制，提高项目履约率。优化投资软环境，提高招商引资的诚信度，统筹和协调园区招商。三是大力拓展经贸市场。加强区域经贸往来，主动承接泛珠三角、长三角等地区的资本、产业、技术、人才等要素的转移。积极引进国外高新技术、先进装备和国内短缺资源，扩大有效进口。巩固亚洲市场，拓展欧美市场，优化出口商品和贸易结构，扩大出口总量，培育自有出口品牌，提高出口商品的档次和附加值。大力支持我市具有比较优势且市场前景好的行业走出去，逐步扩大境外投资。

　　（六）高度关注民生，全面发展各项社会事业。一是优先发展教育和科技事业。以新课程改革为突破口，积极推进素质教育。深化农村教育配套改革，调整城乡中小学布局，优化教育资源配置。发展远程教育和适应产业配套的职业教育。依法引导社会力量办学。发挥高等院校的聚集优势，积极推动高等教育和科技创新的结合。以全国科技进步先进市验收为契机，加大科技投入，大力推进技术创新和成果转化，培育一批具有自主知识产权、高成长性的科技企业。二是推动文化卫生体育等事业发展。积极推进文化体制改革，支持社会办经营性文化产业，加快影视、出版、卡通等文化产业发展。继续送戏、送电影下乡，大力开展广场文化、社区文化、镇村文化等群众性文化活动。创造艺术精品，发展数字电视和移动电视。加快公共卫生体系建设，抓好重大传染病、地方病和职业病的预防控制工作。加快市属医院集团化改革步伐，继续抓好农村新型合作医疗和农村卫生院的建设。继续实施食品药品放心工程。举办长沙市第六届运动会，培育高水平体育竞技表演市场。稳定低生育水平，优化人口结构。三是切实加强劳动就业和社会保障工作。全面落实促进就业和再就业的各项政策，大力发展劳动密集型产业，积极探索多样化的非正规就业形式，鼓励临时性、阶段性、季节性就业。完善城乡就业服务体系，加强职业技能培训和创业培训。建立社会保险扩面征缴长效机制，加强劳动保障和监察工作，依法规范劳动关系，维护劳动者合法权益。今年起实行农村最低生活保障制度，初步构建城乡社会保障体系。

　 （七）加强民主法制建设，着力构建和谐社会。一是发展社会主义民主政治。各级政府要认真执行本级人大及其常委会通过的各项决议、决定，自觉接受人大的法律监督、工作监督。积极支持政协履行政治协商、民主监督、参政议政职能。高标准做好人大代表议案、建议和政协提案的办理和落实工作。定期听取民主党派、工商联、无党派人士和人民团体的意见，诚心接受新闻舆论和社会公众监督。继续完善合村并组工作，依法完成第六次村委会换届选举。加强城市社区建设，完善服务功能。认真完成“四五”普法任务，形成学法守法的社会新风。二是推进创建文明城市工作。以提高人的素质为核心，创建全国文明城市为动力，广泛开展各类文明创建活动，营造“爱我长沙、兴我长沙”的浓厚氛围。创新市民论坛形式，推广社区义工活动，推进文明家庭和社区村组建设。切实加强和改善未成年人的思想道德教育，依法保护妇女、残疾人和未成年人的合法权益，高度重视做好老龄工作。加强全民国防教育和国防后备力量建设，深入开展拥军优属和军民共建活动，密切军政军民关系。三是努力建设和谐长沙。建立更加通畅、更加便利的人民群众反映诉求的渠道，完善公众参与和表达机制，主动及时地协调好各方面的利益和矛盾，形成全市上下统一意志、振奋精神、心情舒畅、团结实干、共图发展的浓厚氛围。深化“平安长沙”创建工作。维护社会公平和正义，正确处理人民内部矛盾。健全信访工作责任制，加强社会治安综合治理，不断提高基层组织化解矛盾的能力。巩固“严打”斗争成果，切实维护国家安全和公共安全。抓好安全生产，严防重特大安全事故的发生，确保社会稳定和安宁。

　　（八）加强政府自身建设，提高领导发展的能力。一是加强制度建设，坚持依法行政。要把建立先进、健全、稳定的制度作为衡量政府治政水平和法治化程度的重要标志。进一步健全重大事项决策论证制度、社会公示和听证制度，继续推行行政问责制，完善经济运行监测和预警体系，改革和完善政府绩效评价制度，从制度上保障和提高政府领导发展的能力。要用制度规范和制约行政权力和行政行为，处理问题，出台规范性文件，要以相应的法律、法规为准绳；治理环境、整顿秩序、行政审批、协调矛盾，要依法依规办事。在任何时候、任何情况下，都不能背离法制的轨道和无视法律的尊严。二是转变政府职能，推进市场化进程。强化政府的宏观管理职能，按照经济社会发展的目标、工作重点，研究和制定战略措施。科学编制“十一五”规划，高质量完成经济普查工作。加快市场取向的改革，凡能由市场调节、企业自主选择或社会中介机构提供服务的事项，政府应坚决退出。要初步建立公共财政体系框架，加强财政资金效益管理，大力降低行政和商务成本。进一步推进政府行政体制改革和管理机制的完善，扩大县（市）经济社会管理权限。继续抓好电子政府建设，推进政务、厂务、村务公开。以完备的基础设施、良好的治理秩序、健全的保障体系，创造更加高效的政务环境，更加开放的市场环境，更加向上的人文环境！三是牢记“两个务必”，树立良好政风。要始终保持谦虚谨慎、不骄不躁和艰苦奋斗的作风，在成绩面前不自满，遇到困难不退缩。大力弘扬求真务实之风，提倡讲真话、办实事、求实效。坚持励精图治，做到发展重于一切，先于一切，不能有丝毫松懈；坚持艰苦创业，做到勤俭节约办事，埋头苦干，不贪图任何虚名；坚持执政为民，做到用权心系人民，正直无私，不让尘埃污染操守。坚决落实党风廉政建设责任制，从严惩治腐败，以良好的政风带出好的民风。时刻教育和鼓励公务员树立群众观念，在事关群众利益的问题上，要立足现实，放眼未来，深谋远虑，既让今人满意，又让后人受惠。决不做图一时之得，损长远之利的事。切实关注民生，竭尽全力地帮助群众解决实际困难。今年要抓好县乡公路建设、农村低保、背街小巷改造、历史区街整修、就业再就业、改善农村办学和医疗卫生条件、加强社会治安和安全生产以及改善城市环境等八件实事，为人民群众带来更加实在的利益。

　　各位代表，长沙的发展和进步已经站在一个新的起点上，让我们在市委的领导下，高举邓小平理论和“三个代表”重要思想伟大旗帜，全面贯彻落实党的十六大和十六届四中全会精神，万众一心、开拓创新、攻坚克难、勇往直前，去描画新的蓝图，创造新的业绩，在建设全面小康社会的征途上实现新的跨越！
